# Podoconiosis treatment in northern Ethiopia (GoLBet): study protocol for a randomised controlled trial

**DOI:** 10.1186/s13063-015-0818-7

**Published:** 2015-07-16

**Authors:** Henok Negussie, Meseret Molla Kassahun, Greg Fegan, Patricia Njuguna, Fikre Enquselassie, Andy McKay, Melanie Newport, Trudie Lang, Gail Davey

**Affiliations:** Brighton & Sussex Medical School, University of Sussex, Falmer, Brighton BN1 9PX UK; College of Development Studies, Addis Ababa University, Addis Ababa, Ethiopia; Department of Statistics, University of Gondar, Gondar, Ethiopia; School of Public Health, Addis Ababa University, Addis Ababa, Ethiopia; Oxford University Centre for Tropical Medicine, Oxford, UK; Department of Economics, University of Sussex, Falmer, Brighton BN1 9SL UK; Kenya Medical Research Institute-Wellcome Trust Research Programme (KWTRP), Kilifi, Kenya

**Keywords:** Podoconiosis, Nonfilarial elephantiasis, Acute dermatolymphangioadenitis, Randomised controlled trial, Neglected tropical disease, Stigma, Disability

## Abstract

**Background:**

Podoconiosis is one of the forgotten types of leg swelling (elephantiasis) in the tropics. Unlike the other, better-known types of leg swelling, podoconiosis is not caused by any parasite, virus or bacterium, but by an abnormal reaction to minerals found in the clay soils of some tropical highland areas. Non-governmental Organizations (NGOs) have been responsible for the development of simple treatment methods without systematic evaluation of its effectiveness. It is essential that a large scale, fully controlled, pragmatic trial of the intervention is conducted. We aim to test the hypothesis that community-based treatment of podoconiosis lymphoedema reduces the frequency of acute dermatolymphangioadenitis episodes (‘acute attacks’) and improves other clinical, social and economic outcomes.

**Methods/Design:**

This is a pragmatic, individually randomised controlled trial. We plan to randomly allocate 680 podoconiosis patients from the East Gojjam Zone in northern Ethiopia to one of two groups: ‘Standard Treatment’ or ‘Delayed Treatment’. Those randomised to standard treatment will receive the hygiene and foot-care intervention from May 2015 for one year, whereas those in the control arm will be followed through 2015 and be offered the intervention in 2016. The trial will be preceded by an economic context survey and a Rapid Ethical Assessment to identify optimal methods of conveying information about the trial and the approaches to obtaining informed consent preferred by the community. The primary outcome will be measured by recording patient recall and using a simple, patient-held diary that will be developed to record episodes of acute attacks. Adherence to treatment, clinical stage of disease, quality of life, disability and stigma will be considered secondary outcome measures. Other outcomes will include adverse events and economic productivity. Assessments will be made at baseline and at 3, 6, 9 and 12 months thereafter.

**Discussion:**

The evidence is highly likely to inform implementation of the new master plan for integrated control of Neglected Tropical Diseases (NTDs), in which podoconiosis is identified as one of eight NTDs prioritised for control. Potentially, an estimated 3 million patients in Ethiopia will therefore benefit from the results of this trial.

**Trial registration:**

International Standard Randomised Controlled Trial Number. Registration number: ISRCTN67805210. Date of registration: 24 January 2013.

## Background

Podoconiosis is a form of lymphoedema (leg swelling) arising in people going barefoot in highland tropical areas [1]. It is a significant problem in tropical Africa where irritant soils have been generated by environmental conditions of high altitude (>1,000m) and high rainfall (>1,000mm per annum), and are farmed by very poor people who cannot afford shoes or water for washing [2]. Although rarely a direct cause of mortality, it disables an estimated 4 million subsistence farmers in tropical Africa, greatly reducing productivity [3], leading to significant stigma from the community [4–6] as well as health professionals [7], and low quality of life [8]. Despite the high impact of podoconiosis on rural farming communities, treatment has been hampered by misdiagnosis (chiefly confusion with filarial lymphoedema) and fatalism [7]. Only recently has any form of treatment been offered to people with podoconiosis, and this has grown up from grassroots level without systematic evaluation.

At present, foot hygiene services are offered to a total of about 60,000 patients by non-government organisations in three regions in Ethiopia. While one non-controlled follow-up study has assessed clinical and quality of life changes [9], the impact of the intervention has not yet been evaluated through a formal trial. It is important that fully controlled evaluation with measurement of a wider range of outcomes now happens, before the intervention is expanded to an additional 300 *woredas* (districts) in which podoconiosis was found to be prevalent in a recent nationwide mapping [10]. The evidence generated from this trial will be conveyed through a dissemination workshop to key government and non-government stakeholders once analysis is complete and is highly likely to inform implementation of the new master plan for integrated control of Neglected Tropical Diseases (NTDs), in which podoconiosis is identified as one of 8 NTDs prioritised for control. Potentially, an estimated 3 million patients in Ethiopia will therefore benefit from the results of this trial.

### Objectives

The primary objective of the trial is to test the hypothesis that community-based treatment of podoconiosis lymphoedema reduces the frequency of acute dermatolymphangioadenitis episodes (ADLA, ‘acute attacks’). The secondary objective is to test the hypothesis that community-based treatment of podoconiosis lymphoedema improves other clinical, social and economic outcomes.

### Endpoints

The endpoints for this trial are listed in Table 1.

**Table 1:** Endpoints

	Endpoint details
Primary endpoint	Cumulative incidence of ADLA (total number of incident episodes over the 12-month follow-up period).
Secondary endpoints	Adherence to treatment
	Foot washing, use of ointment, use of bandages, elevation, exercises, use of socks and shoes.
	Clinical outcomes:
	Clinical stage of disease^a^
	Lower leg and foot circumferences^b^
	Presence of mossy changes
	Wounds and inter-digital lesions;
	Duration of ADLA days
	Social outcomes:
	Quality of life^c^
	Perceived stigma^d^
	Economic outcomes:
	Economic productivity^e^

ADLA, acute dermatolymphangioadenitis

^a^Using a scale specifically developed for use in podoconiosis patients [22]

^b^Measured in centimetres at mid-calf and mid-foot

^c^Using validated Amharic translation of Dermatology Life Quality Index [23] and WHO Disability Assessment Schedule-II (WHODAS-II)

^d^Using scale developed to measure stigma among podoconiosis patients (24)

^e^Other measures of economic vulnerability found to be related to podoconiosis in economic context survey

## Methods/Design

### Trial design

This is a pragmatic Randomised Controlled Trial (RCT) with two arms that will compare podoconiosis lymphoedema management in the community with delayed treatment (as the control).

### Study setting

The study will be based at the recently founded International Orthodox Christian Charities (IOCC) Debre Markos Podoconiosis Project [11] in northern Ethiopia. The protocol will be carried out in Aneded *woreda*, with an estimated total population of 89,446 [12], and made up of 19 *kebeles* (smallest administrative sub-unit) each with an average population of approximately 5,000 people and two Health Centres.

### Participants

Participants will be drawn from adults aged 18 years and older registered as affected with podoconiosis in Aneded *woreda*, East Gojjam Zone. These patients are representative of all those in Amhara regional state in northern Ethiopia, where the prevalence of podoconiosis is estimated as 3.4 % in the adult population [13]. Until 2010, there was no government or private treatment for podoconiosis anywhere in the region. In 2010, a podoconiosis project was established by IOCC and began treatment of 200 patients. To date, approximately 1,000 patients have been treated, and thousands more are on the waiting list for treatment [13]. Podoconiosis patients from nine *kebeles* of Aneded *woreda* will be invited to participate. Diagnosis of podoconiosis is currently based on clinical features: history of aching and burning starting in the feet, swelling progressing from the forefoot up the leg; patients must have lived for at least 10 years in communities 1,500 meters above sea level; and filarial lymphoedema must have been excluded (negative Binax-Filariasis rapid antigen test). Among people with lymphoedema in areas in which mass drug administration for lymphatic filariasis has never occurred, a negative rapid antigen test has high predictive value for podoconiosis.

### Eligibility criteria

The Inclusion, exclusion and withdrawal criteria are presented in Table 2.

**Table 2:** Inclusion, exclusion and withdrawal criteria

Inclusion criteria	
1.	Be at least 18-years old
2.	Have provided informed consent
3.	Have a diagnosis of at least Stage 2 podoconiosis (that is, podoconiosis lymphoedema) confirmed by the trial team
4.	Have a negative ICT card test
5.	Intends to remain within the area during the study period
Exclusion criteria	
1.	Already undertaking self-treatment comparable to the intervention
2.	Nodular disease preventing use of shoes (will be referred for nodulectomy)
3.	Complex wounds (will be referred for specialist care in Debre Markos Hospital)
4.	Patient has a history of allergic reaction to treatment materials
5.	Mental health or learning disorder affecting ability to adhere with treatment
6.	Physical disability beyond podoconiosis precluding attendance at group sessions
7.	Disease considered by the Trial Co-ordinator to affect ability to self-treat.
Withdrawal criteria	
1.	Wishes to withdraw consent to participate in trial
2.	Move outside study *Woreda*
3.	Experiences of SAEs considered by trial coordinator to compromise ability to participate
4.	Experiences of SUSAR

### Sensitisation and recruitment procedures

The Rapid Ethical Assessment (REA) has been used prior to other studies to establish the most appropriate way of working with podoconiosis communities in southern Ethiopia [14] and Cameroon, and we plan to use this method in Gojjam before the trial. Rapid Ethical Assessment is a form of ethnographic assessment through which information about a community’s views and perspectives on specific research may be elicited and used to inform, for example, development of sensitisation approaches or consent processes [15]. Our sensitisation and recruitment will be modelled on the results of the REA, and the format of Information Sheets and Consent Forms will depend on responses given by participants in the REA. Written informed consent will be obtained from each participant.

### Community engagement strategy

The clinical service arm of the IOCC Podoconiosis Project is complemented by extensive community education and mobilisation aimed at decreasing misconceptions about disease aetiology and transmission, and stimulating awareness of ways of preventing and treating podoconiosis [11]. In addition, a community sensitisation workshop involving political, community and religious leaders, government representatives and local health workers will be conducted. These groups will play an important role in disseminating information about the trial and will act as the first level of ‘gatekeeping’ to the participants. Engagement with the community will continue throughout the trial via the Patient Associations (PAs).

### Sample size

We calculated the required sample size based on a mean (Standard Deviation) baseline incidence of ADLA of 5.6 (4.9) episodes/year reported in a survey conducted in November 2011 in this population [11]. We assumed a reduction in ADLA frequency of 28 % (from 5.6 to 4.0 episodes per year). Based on a sample size calculation for comparison of two means [16], a 90 % power, an α-level of 0.05, and a 40 % increase to enable adjustment for four confounders, and 15 % drop-out rate [9], that is, adjusting the sample size per group (206 × 1.4/0.85), we would need a total 680 patients: 340 patients in the immediate treatment arm and 340 patients in the control arm.

### Randomisation

Initial community sensitisation through *woreda-* and *kebele-*level officials will be followed by Health Extension Workers enumerating patients in their respective *kebeles* selected for the study in the *woreda*. All patients identified by Health Extension Workers (HEWs) will be visited at home by a data collector and consent requested for preliminary evaluation against the inclusion and exclusion criteria. The data collector will record potential willingness to take part in the trial, Geographic Information System (GIS) coordinates of the house and local contact information (for example, *kebele* office, nearest mobile phone owner). Patients who appear to be eligible will then be given an appointment date for enrolment at the nearest health facility. At enrolment, all patients will be asked for full consent for the trial; be evaluated against inclusion and exclusion criteria once more, and ICT tests and baseline measurements will be carried out. Community Podoconiosis Assistants (CPAs), Data Collectors (DCs) and data supervisors assisted by the study coordinator and data manager, will enrol patients.

Data collectors will then map these patients by recording Geographic Information System (GIS) coordinates of their houses, generating a target list of approximately 850 potential trial participants and take consent to examine each listed patient. If more than one eligible patient is found in one household, one will be randomly selected using a lottery method. Using a sample frame of households within *kebeles*, a randomisation list will be prepared by statisticians at Kenya Medical Research Institute-Wellcome Trust Research Programme (KWTRP). Patients will be randomised in excess of the required sample size to allow for those who decline the invitation to participate or who are later found not to fulfil the inclusion criteria.

### Blinding

Given the nature of the intervention, the trial cannot be blinded. Although data collectors will be independent of those delivering treatment, they will be able to deduce the randomization status of participants when they see the supplies required for treatment. Similarly, statisticians conducting the analysis cannot be blinded. To minimise bias, an *a priori* analytical plan will be defined as the intervention is in progress and will be adhered to.

### Study visits

A timeline of the study is presented in Table 3.

**Table 3:** Study timeline

	Before enrolment	Enrolment	Baseline	Visit 1	Visit 2	Visit 3	Visit 4 close-out
Timepoint		*-t*_*1*_	0	*3 months*	*6 months*	*9 months*	*12 months*
Screening	X						
GIS mapping	X						
Potential willingness to participate	X						
Inclusion and exclusion criteria	X	X					
Consent		X					
Randomisation			X				
Socio-demographic			X				X
Economic		X	X		X		X
ADLA (recall)		X	X				
ADLA diary				X	X	X	X
SAEs			X	X	X	X	X
DLQI			X	X	X	X	X
WHO-DAS II			X		X		X
Stigma scale			X		X		X
Clinical stage			X	X	X	X	X
Mossy lesions			X		X		X
Foot and leg Circumference			X		X		X
Interdigital lesions			X		X		X
Foot care and hygiene intervention (immediate treatment group)*							

*Monthly intervention meetings

### Intervention

The intervention in this trial is podoconiosis lymphoedema hygiene and foot-care management. This consists of monthly group meetings with instruction and practical demonstration of foot hygiene and foot care by Community Podoconiosis Assistants (CPAs), supporting daily self-treatment. In outline, ‘foot hygiene’ comprises soaking feet, washing with soap, rinsing with clean water, drying and application of emollient. ‘Foot care’ includes supervised use of single-layer, non-elastic bandages for disease stages ≥3; foot and calf exercises; instruction to practice foot hygiene daily at home; instruction to elevate the foot of the bed or areas slept on; instruction to use socks and shoes during waking hours. Those randomised to immediate treatment will receive a hygiene and foot-care intervention from December 2014 for one year, while those randomised to the control arm will be followed through 2014 and will be offered the intervention in 2015, unless it has been shown to be ineffective or harmful.

The trial interventions are described in Table 4.

**Table 4:** Trial intervention

Action	Necessary items
Soak for 15 minutes	Bowl of water
Wash with soap	Named brand (GIV international white)
Apply emollient	Each patient will be issued with Whitfield’s ointment (2 tubes of 20 gm for a period of 1 month)
Apply bandages	For stage ≥3 disease; two short-stretch (<80 % stretch) bandages, spiral from foot, overlap 50 %, continue 8-10 cm above oedema
Elevation and exercises	Ankle rotation exercises.
	Elevate foot end of sleeping mattress, if used
Use socks and shoes	Temporary (tyre) shoes if bandages issued
	One pair robust, closed shoes and two pairs of socks after 3 months of treatment

Each patient randomised to the treatment arm will be allocated one bar of toilet soap (GIV international white) every month, and rate of use will be monitored at monthly meetings. In addition, patients will have dispensed 40 gm of Whitfield’s ointment sufficient for daily application for one month, will be asked to bring used tubes with any remaining ointment to their group meeting each month, and excess ointment will be recorded. Patients with stage 3 and greater podoconiosis will each receive two short-stretch (<80 % stretch) bandages for each affected foot, one to be worn while the other is being washed and dried. Under likely home conditions, it is anticipated that bandages will need replacement after approximately 6 months. However, the condition of the bandages will be checked at monthly group meetings, and replacements given as required. Patients receiving bandages will also be given temporary, loose-fitting shoes at the enrolment visit. All patients’ feet will be measured at 3 months (once the majority of the swelling is anticipated to have subsided), and they will be given a pair of strong, closed ‘trainer’ or leather shoes and two pairs of socks. Patients will be encouraged to wear shoes all day, every day, no matter what their activities, and will be shown how to clean and care for their shoes and socks. We anticipate that high quality shoes will last for the full year of the trial, while socks will require replacement after 6 months, but the condition of shoes and socks will be monitored through the monthly meetings. Soap, Whitfield’s ointment and bandages will be kept out of the reach of children in a plastic treatment box. Standard operating procedures fully describe the hygiene products, their storage and application to assure consistency between participants, healthcare workers and study visits.

### Outcomes and measurement

The primary outcome is the total number of ADLA episodes during the 12-month follow-up period. ADLA episodes have been demonstrated to be a common complication of podoconiosis lymphoedema, occurring on average five times per year, and contributing to an average 4.4 days off work per episode [13, 17]. They are highly distinctive to patients, who in the study area, refer to these episodes as ‘*michader*’ (‘the blight that casts you down causing you to spend the night where it hit you’). Patients describe pain (in inguinal nodes or in the lower leg), swelling in addition to the ‘normal’ lymphoedema, and redness of the leg (sometimes as discrete longitudinal lines), chills (‘*birrd*’), anorexia and sometimes lower leg skin peeling towards the end of the episode. Considering most of the patients are unable to write, the trial team have designed the diary such that each patient marks a check mark (✓) or a dash (-) under the boxes corresponding to drawings showing a healthy patient (working in the field) and a patient experiencing acute attacks (shown in bed) for each day of the month. During the pilot phase, patients had no difficulty in marking the diaries based on their experiences of acute attacks. For the trial, patients will be trained to complete diaries at the outset and those who may have difficulty (including the elderly or visually impaired) will be requested to have their diaries filled by family members or neighbours who are able to read and write. In addition, diaries of patients in the immediate treatment group will be checked and collected at every monthly intervention meetings, whereas all diaries of patients in the control group will be checked for the first month and a random 10 % on subsequent months. Data collectors will discuss these diaries with patients at the quarterly data collection visit at home, and discrepancies between diary and recall will be reconciled.

All secondary outcomes will be measured following internationally validated scales where possible. Where no internationally validated scale is available, previously reported questionnaires will be used (Table 1).

### Safety

This is a pragmatic trial of hygiene and foot-care interventions that use household and community care-based products that are not prescription medical products and thus are associated with few safety concerns. Nonetheless, safety will be carefully monitored to confirm that there is indeed minimal risk. Adverse events in both arms will be reported on the case record forms completed by the data collectors at the quarterly home visits. Adverse events related to the intervention will be actively elicited from those randomised to immediate treatment by CPAs at the monthly meetings via specific questions. The study coordinator will then confirm occurrence of events, analyse them and report them by telephone and email within 24 hours to the Local Safety Monitors (LSMs), and the investigators who will reach a consensus on whether a Serious Adverse Event (SAE) needs further defining as a Suspected and Unexpected Serious Adverse Reaction (SUSAR) based on the type of event, the relationship of the event to the time of administration and the known biology of intervention. However, adverse events, regardless of relation to the intervention, will receive treatment accordingly. Any SUSAR will then be reported within 24 hours of detection to the Trial Steering Committee, Institutional Review Boards (IRBs), ethics committees and regulatory authorities in Ethiopia and the United Kingdom.

### Adverse event monitoring

We conducted a risk assessment for this trial and considered whether a Data Safety Monitoring Board (DSMB) was required. The reason for putting a DSMB in place is to monitor safety and efficacy outcomes and consider whether the trial needs to be revised or stopped early. We concluded that safety events are too unlikely to indicate that a DSMB should monitor events. On considering efficacy and whether an interim analysis would be appropriate to assess whether the question has been answered earlier than predicted, we also concluded this was unnecessary. We consider that ending the trial early following a DSMB assessment of an interim analysis would be essentially meaningless in this setting because as of yet, this intervention is not available to the population, and therefore, we would be better continuing the trial to its full power in order to obtain a full data set to support analysis of the primary and secondary endpoints. Therefore, based on recommendations in articles on when a DSMB is required, we have put local independent safety monitors in place who will review all SAEs immediately on being notified by the study team and review all adverse events monthly [18, 19].

We anticipate that the hygiene and foot-care intervention will bring clinical improvements to the lower legs and social and quality of life changes to the patient as a whole. These will be recorded as part of the assessment of scientific objectives. Symptom enquiry related to the primary outcome (ADLA) will also create the opportunity to identify unexpected systemic effects of the intervention.

The standard of comparison or the control arm is delayed intervention. If the trial shows benefit with this intervention, the data will then be used to influence policy in Ethiopia, and elsewhere, to make this intervention widely available.

### Assessment of quality

The trial will be monitored by staff at the KEMRI-Wellcome Trust Programme’s Kilifi Clinical Trials Facility (KCTF), who will perform on-site and database monitoring. The approach for trial monitoring will include mentoring and supporting the trial staff and healthcare workers, working closely with them before, during and after the trial to help achieve an ethical, safe and accurate trial that answers the questions set within this protocol.

Pre-study start site visits will occur before the first participant is enrolled. Standard Operating Procedures (SOPs) will be developed followed by a study initiation visit that will be conducted by the study coordinator and supported by the monitoring staff from Kilifi. Here, the SOPs will be used to ‘walk’ through the study with everyone involved. All trial staff will be trained and certified for Good Clinical Practice (ICH-GCP). A trial monitoring plan will be written and signed off by the Trial Steering Committee. This will detail how many monitoring visits are planned and specify the key data points that require checking against source data. The fact that the products being used are known and safe, the trial is essentially open and there are no plans for interim analyses, internal monitoring being performed by KCTF staff [20] is in accord with WHO guidance [21].

### Data management

All protocol required information will be collected in Case Report Forms (CRF) designed by the investigators and KCTF. Data will be collected on paper proformas, will undergo a quality control step, and then be transcribed direct onto the trial data management system, OpenClinica. A random 10 % subset will be checked by the Ethiopia-based Data Manager. Data will be backed up daily onto secure external hard drives, and uploaded to the OpenClinica server at weekly intervals. Data management will be performed by the CTF in Kilifi, who operate an online OpenClinica data management system (Akaza Research, Waltham, MA). The trial monitor will visit for source data verification.

### Statistical methods

Statistical support will be provided by the KEMRI-Wellcome Trust Programme in Kilifi, Kenya. A detailed Statistical Analysis Plan will be developed in 2014-15 while the intervention is in process, and will be approved by the Trial Steering Committee. The main analysis will be a test of mean number of ADLA events by each arm over the observation period.

### Ethical approval

The study is approved by the Research Governance and Ethics Committee of the University of Sussex (approval number 13/107/DAV; approval date: 12/08/2014), College of Health Sciences, Addis Ababa University (meeting number 056/2014; protocol number 071/13/SPH; approval date: 12/01/2014), the Food, Medicine and Health Care Administration and Control Authority (reference number 02/6-1/05/39; approval date: 09/04/2014) and the National Research Ethics Review Committee of the Ministries of Health and Science and Technology in Ethiopia (approval number 3-1/794/06; approval date: 02/06/2014).

## Discussion

Limited evidence exists for the effectiveness of lymphoedema care similar to that used for filarial lymphoedema. To date, one non-controlled follow-up study among podoconiosis patients indicated improvements in quality of life measures very rapidly (within the first 3 months), but slower clinical changes (leg circumference and stage of disease), with statistically significant changes being observed after 6-9 months of follow-up [9]. However, there are no published controlled clinical trials investigating the effectiveness of lymphoedema management in podoconiosis. We have therefore designed a full-scale trial to interpret the results to understand the timing of clinical changes and their relationship to psychosocial changes in order to be able to advise the optimal duration of supervised treatment. Our study will be the first fully controlled, pragmatic trial of the intervention, with measurement of a wider range of outcomes, including frequency of acute dermatolymphangioadenitis (ADLA) in podoconiosis lymphoedema. The evidence is highly likely to inform implementation of the new master plan for integrated control of Neglected Tropical Diseases (NTDs), in which podoconiosis is identified as one of eight NTDs prioritised for control. Potentially, an estimated 3 million patients in Ethiopia will therefore benefit from the results of this trial.

## Trial status

The Rapid Ethical Assessment, the economic context survey and validation of the patient-held diary have all been completed. Recruitment for the trial commenced on 8 October 2014.

## Abbreviations

ADLA: acute dermatolymphangioadenitis
CRF: case report form
CPA: Community Podoconiosis Assistant
DLQI: Dermatology Life Quality Index
DSMB: Data Safety Monitoring Board
ICH-GCP: International Conference on Harmonisation - Good Clinical Practice
IOCC: International Orthodox Christian Charities
KCTF: Kilifi Clinical Trials Facility
KWTRP: Kenya Medical Research Institute-Wellcome Trust Research Programme
LSM: Local Safety Monitor
NTD: Neglected Tropical Diseases
NGO: non-governmental organisation
REA: Rapid Ethical Assessment
SAE: serious adverse event
SUSAR: suspected and unsuspected serious adverse reaction
WHODAS: World Health Organisation Disability Assessment Schedule
